# Retraction Note: A numerical study of chemical reaction in a nanofluid flow due to rotating disk in the presence of magnetic field

**DOI:** 10.1038/s41598-023-34630-w

**Published:** 2023-05-10

**Authors:** Muhammad Ramzan, Noor Saeed Khan, Poom Kumam, Raees Khan

**Affiliations:** 1 KMUTTFixed Point Research Laboratory, Room SCL 802 Fixed Point Laboratory, Science Laboratory Building, Department of Mathematics, Faculty of Science, King Mongkut’s University of Technology Thonburi (KMUTT), Bangkok, 10140 Thailand; 2 Center of Excellence in Theoretical and Computational Science (TaCS-CoE), Science Laboratory Building, Faculty of Science, King Mongkut’s University of Technology Thonburi (KMUTT), 126 Pracha-Uthit Road, Bang Mod, Thung Khru, Bangkok, 10140 Thailand; 3 Division of Science and Technology, Department of Mathematics, University of Education, Lahore, 54000 Pakistan; 4 Department of Medical Research, China Medical University Hospital, China Medical University, Taichung, 40402 Taiwan; 5 Department of Mathematics, FATA University, Darra Adam Khel, FR Kohat, Pakistan

Retraction of: *Scientific Reports* 10.1038/s41598-021-98881-1, published online 29 September 2021

The Editors have retracted this article.

After publication concerns were raised about the authorship of this article. Investigation by the Editors has found evidence of manipulation of the authorship during the peer review process. As the authorship could not be verified the Editors no longer have confidence in the content of this article.

Noor Saeed Khan disagrees with this retraction. Muhammad Ramzan, Poom Kumam and Raees Khan have not responded to correspondence from the Editors about this retraction.

